# The impact of social support and stress on academic burnout among medical students in online learning: The mediating role of resilience

**DOI:** 10.3389/fpubh.2022.938132

**Published:** 2022-07-22

**Authors:** Yue Liu, Zhe Cao

**Affiliations:** ^1^School of Education Science, Nanjing Normal University, Nanjing, China; ^2^Department of Cardiology, The Second Affiliated Hospital of Nanjing Medical University, Nanjing, China

**Keywords:** stress, social support, resilience, academic burnout, medical students, online learning, COVID-19

## Abstract

**Background:**

As the COVID-19 pandemic continues, online learning and long-term isolation from social and clinical settings has exacerbated mental health problems and symptoms of academic burnout among medical students. However, few studies have discussed symptoms of academic burnout as a result of reduced social support, and increased stress among medical students during the process of online learning. To fill this gap, this study investigated the influencing factors and mechanism of academic burnout in medical students' online learning process. Both the positive inhibition effect of positive factors such as social support, and the negative aggravation effect of negative factors such as stress were explored, while the mediating and protecting role of resilience is also discussed.

**Method:**

We collected survey data from a total of 817 medical students from a medical school in China who participated in online learning during the fall 2021 semester. An online questionnaire was sent to the students in January, 2022. Items adapted from the DASS Scale developed by Lovibond and Lovibond were used to measure medical students' stress levels. The perceived social support of medical students was assessed by the Gregory MSPSS. Resilience was evaluated by the 10-Item Connor–Davidson Resilience Scale (CD-RISC). Items from the Maslach Burnout Inventory–Student Survey (MBI-SS) were used to calculate students' academic burnout. Descriptive analysis, correlation analysis, hierarchical linear regression analysis and structural equation modeling were used to analyze the collected data.

**Results:**

The results identified that in the context of online learning there was a positive correlation between medical students' stress and academic burnout, and their resilience played a partial mediating role. However, social support did not directly affect academic burnout, but inhibited the prevalence of academic burnout through resilience. In addition, stress was negatively related to resilience, while social support was positively related to resilience. Resilience was found to be negatively related to medical students' academic burnout in online learning.

**Conclusion:**

The results of this study can provide a reference for the future development of appropriate educational strategies and coping measures to ameliorate the academic burnout of medical students.

## Introduction

Since the COVID-19 outbreak, online learning has become an expanded form of learning on campus in response to sudden lockdowns. Meanwhile, the COVID-19 pandemic also has profound impacts on medical students' learning, with the majority of medical students experiencing online learning (1). The advantage of online learning is flexibility without the limitation of time and place, which ensures the orderly development of teaching, and plays an important role in ensuring that students complete theoretical courses (2). At the same time, online learning poses many challenges to education systems, and has an especially negative effect on medical students (3).

The most significant challenge is the poor learning outcomes of online learning for medical students, such as decreased academic performance and low learning satisfaction (4). What is more, a survey of medical students' online learning during the COVID-19 pandemic suggested that nearly half of medical students were experiencing academic burnout (5), which is closely related to their mental health status (6).

Medical students reported higher levels of academic burnout and showed more signs of stress, anxiety, and depression than students in other majors. The COVID-19 pandemic has exacerbated the poor mental health of the medical student population (7), particularly the stress of medical students (8). Through a comparative study of 764 medical students before and after the COVID-19 pandemic, Li et al. (9) found that medical students were experiencing increasing stress during the COVID-19 pandemic. Prior research explored the mental health problems of medical students, and discussed social support at the same time (10, 11). During the COVID-19 pandemic, “physical isolation” was implemented to prevent infection. As a way of isolating teachers and students, there was a transition from classroom learning to virtual learning, which could lead to individual isolation and lack of social support (12).

Stress was identified as a decisive risk factor for burnout, while social support was regarded as a protective factor for burnout in prior studies (13). The burnout of medical students, as future doctors, will affect public health (14). Meanwhile, it has been confirmed that stress (15) and perceived social support (16) of medical students also had an influence on the development of resilience, which is a protective mechanism against burnout (17–19).

The mounting evidence confirmed the independent correlation between stress, social support, resilience and academic burnout, as well as the mediating effect of resilience on job stress and burnout (20, 21). However, there are the following deficiencies: firstly, the role of resilience in the relationship between social support and academic burnout remains unknown; secondly, the previous research on stress and the resilience of medical students was mostly based on clinical training and campus learning, or was to explore the problems of medical students' mental health during the COVID-19 pandemic. However, research probing the possible relationship between stress, social supports, resilience, and academic burnout among medical students in the context of online learning during the COVID-19 pandemic is rare.

Since the outbreak of the COVID-19 pandemic, there has been increasing evidence reported that online courses which cannot provide practical and experimental opportunities for medical students has led to academic burnout and stress among medical students (22). Resilience, as a positive psychological resource, should be studied as a protective mechanism for the mental health of medical students to reduce losses in the field of medical education during the COVID-19 and post-COVID-19 era. Therefore, the purpose of this study is to explore the relation between stress, social support, resilience and academic burnout, especially the mediating role of resilience among medical students in online learning during the COVID-19 pandemic.

## Theoretical background and hypotheses

### Stress, social support, and academic burnout among medical students

Burnout, which is a serious issue in the public health area, can be characterized as emotional exhaustion, cynicism, and low personal efficacy (23, 24). In this study, we focus on academic burnout in the medical student population, which has a higher rate of burnout symptoms than other populations (25). Differing from job burnout, academic burnout emphasizes that students are exhausted due to learning demands, have a cynical attitude toward learning, and have a low sense of learning achievement as students (26). A number of studies have shown that medical students who suffer from academic burnout have a higher level of stress (27–29). Stress is defined as the transactional process that occurs when an event is perceived to be relevant to an individual's well-being, has the potential to cause harm or loss, and requires mental, physical and/or behavioral efforts to manage the event and its outcome (30). The outward manifestations of stress are difficulty in relaxing, irritability, nervous excitability, and impatience (31). Stress plays an important role in the overall mental health and academic performance of medical students. Students who are stressed or under a high degree of stress for long periods of time show poor academic performance and mental health problems such as anxiety and depression (32, 33). Often the stress has a negative impact on individual studies or life. A significant correlation has been found between stress and academic burnout among medical students. For instance, through a cross-sectional study of 241 medical students, Yusoff et al. (34) summarized that neuroticism, emotional intelligence, and stress negatively predicted academic burnout, that is, stress should be considered as a risk factor of academic burnout among medical students.

Therefore, according to the important connection between stress and burnout mentioned above, it is necessary to investigate both of these two factors among medical students in online learning. Specifically, researchers have found that medical students experienced a higher degree of stress in online learning than prior school learning during the COVID-19 pandemic (7, 35), and it is worth our attention whether such stress will lead to a higher degree of academic burnout among medical students in online learning during the COVID-19 pandemic. Thus, we proposed the following hypothesis:

H1a: Stress is positively correlated with academic burnout among medical students in online learning during the COVID-19 pandemic.

Online learning during the COVID-19 pandemic has been implemented to control the spread of infection. The transition from classroom learning to virtual learning isolated medical students from campus, clinical settings, teachers, and friends, and may have led to personal isolation and a lack of social support (11, 12). Social support, which is considered as an important factor in an individual's mental health, refers to the psychological or physical help provided by family, friends, and others to an individual facing difficulties (36). Different from the role of stress, social support is a protective factor for the academic burnout of medical students. For instance, perceived social support was established as a protective factor for effective coping with three domains of burnout in Kilic et al.'s (13) study. A meta-analysis of the relation between social support and students' burnout indicated that social support, especially school and teacher support, has a strong negative relationship with student burnout (37). Thus, medical students are spatially isolated from school and teachers during COVID-19, and the impact of reduced social support on academic burnout deserves our attention. Therefore, we proposed that:

H1b: Social support is negatively correlated with academic burnout among medical students in online learning during the COVID-19 pandemic.

### The mediating role of resilience between stress-social support and academic burnout

Resilience can be described as a relatively good psychological consequence of coping with challenges, adversities, and other adverse events (38, 39). The concept of resilience indicates the reason why individuals with high levels of stress can also thrive and gain a higher level of competence to cope with challenges. Resilience is thought to be a resource that individuals use to resist stress, and to cushion the negative effects of stress in some studies. Bajaj et al. (40) verified that there is a negative correlation between stress and resilience among undergraduate students. Shi et al. (41) showed that reducing the perceived stress of medical students can enhance their resilience. Thus, we proposed that:

H2a: Stress is negatively correlated with resilience among medical students in online learning during the COVID-19 pandemic.

In contrast to stress, social support has been proved to be an effective mechanism for improving individual resilience in several studies. For instance, Ozsaban et al.'s (42) research demonstrated that nursing students with high levels of psychological resilience perceived higher levels of social support, while Goulet et al. (43) indicated that female college students with higher levels of social support showed higher levels of resilience than those with lower levels of social support. Thus, we proposed that:

H2b: Social support is positively correlated with resilience among medical students in online learning during the COVID-19 pandemic.

Resilience is considered to be a protective mechanism against the consequences of burnout in several studies. For example, Guo et al. (44) found that resilience was an important predictor of burnout among nurses. Houpy et al. (45) indicated that the resilience of medical students is lower than that of the general population sample, and that resilient students did not experience symptoms of burnout and were able to deal with difficult clinical events well. Thus, we hypothesized that:

H3: Resilience is negatively correlated with academic burnout among medical students in online learning during the COVID-19 pandemic.

As for the mediating role of resilience, some evidence has been provided by several studies. For example, Janus et al. (46) indicated that the relationship between stress and depressive symptoms was weakened for students with high resilience. In Kaplan et al.'s research (47), the relationship between mindfulness and burnout was partially mediated by resilience. Hao et al. (20) found that resilience can prevent burnout from developing by relieving stress among civil servants in China. Based on the existing literature, it can be concluded that the impact of mental health factors including stress on burnout is mediated by resilience. Thus, we proposed that:

H4a: The link between stress and academic burnout is reduced when the mediating variables of resilience are controlled.

Besides, the mediating role of resilience on the association between social support and loneliness (48), mental well-being (49), and sleep quality (50) was proved. Nevertheless, direct evidence that resilience is the mediator in the relationship between social support and burnout has not been provided by the existing literature. Both social support and stress are predictors of resilience and burnout. It is also worth exploring whether the impact of the sharp decrease in perceived social support on burnout is affected by resilience. Stress is a risk factor for academic burnout, while social support is a protective factor for academic burnout. In addition to exploring the influence mechanism of “stress-resilience-academic burnout,” it is also worth exploring whether the impact of the sharp decrease in perceived social support on the burnout of medical students is affected by the mediating effect of resilience during the COVID-19 pandemic. Furthermore, Meneghel et al. (51) confirmed that job social resources had an impact on team resilience, and in turn affected performance. According to the Job-Demand Resources Model, social support is an important job resource; therefore, we can speculate that resilience plays a mediating role in the relationship between social support and medical students' negative performance, that is, academic burnout. The results can offer suggestions for providing effective and appropriate social support for future medical students when facing stressful situations with high resilience for avoiding burnout. Thus, we proposed that:

H4b: The link between social support and academic burnout is reduced when the mediating variables of resilience are controlled.

### Hypothesized conceptual model

**Figure 1:**
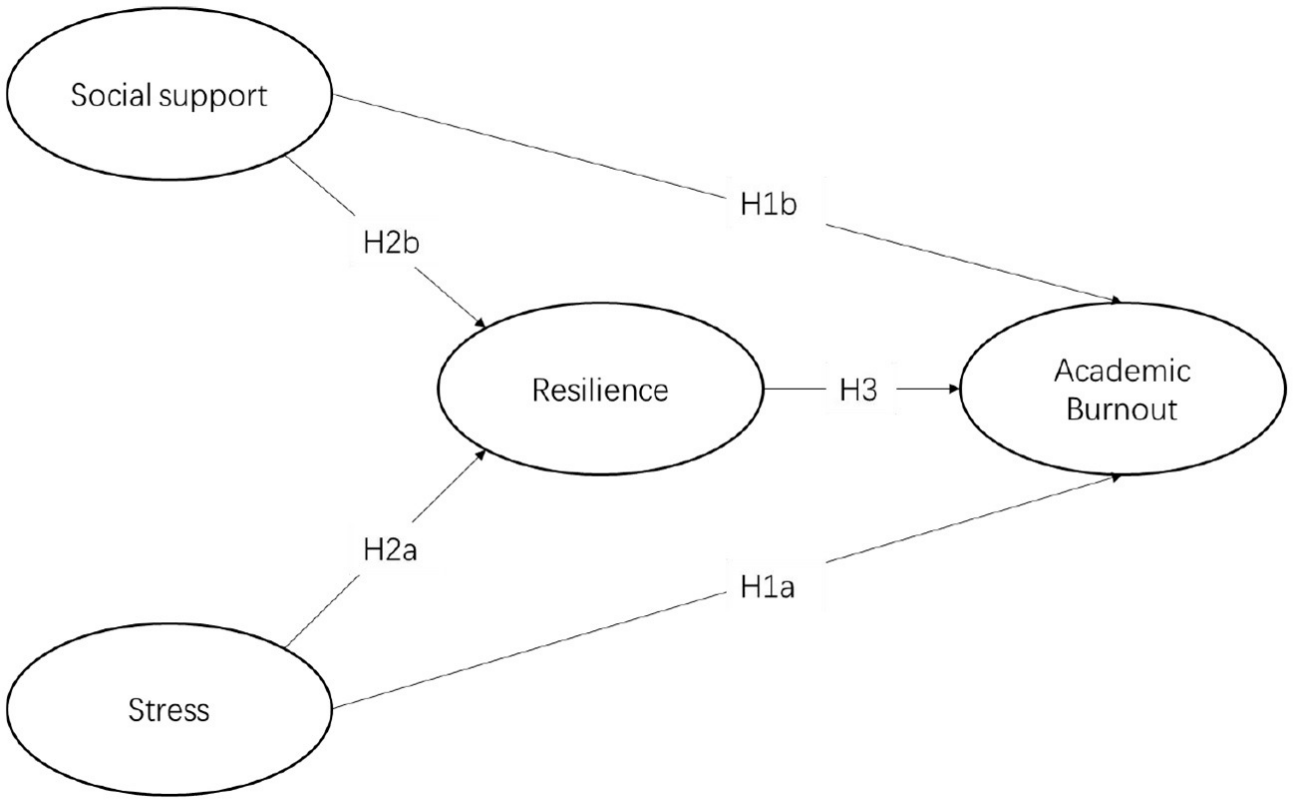
Hypothesized model.

The Job-Demand Resources Model (JD-R model) proposes that the work characteristics of all jobs can be summarized as job demands and resources which include stress, social support, and so on (52). During the COVID-19 pandemic, medical students, as future doctors, were under stress to learn online, which can drain their mental resources and lead to burnout (53). Social support can then serve as job resources to help medical students resist burnout (54). The mediating effect of resilience on the relationship between job demands and workers' performance, as well as job resources and workers' performance has been explored in prior studies. For example, Ceschi et al. (55) confirmed the moderating role of resilience in the mediating influence mechanism of resilience on the relationship between job demands and task performance based on the Job-Demand Resources Model. Therefore, this study speculated that the resilience of medical students may alleviate the burnout caused by the stress of job demands, and may also play a mediating role in the negative correlation between social support and burnout. Based on the above theoretical background and the JD-R model, this study explored the relationship between medical students' stress, social support, and academic burnout, as well as the mediating role of resilience among them. Five hypotheses and the hypothesized conceptual model of this study were proposed, as shown in Figure 1.

## Methodology

### Participants and procedures

A total of 817 medical students from a medical college in Nanjing, China, who had previously studied online at home due to the COVID-19 pandemic during the fall 2021 semester, were recruited. All the participants completed anonymous online surveys *via* Questionnaire Star (https://www.wjx.cn/) which is widely used as a professional online survey tool in China. In order to ensure the reliability of the research results, ethical considerations were followed to conduct the study. The purpose and voluntary nature of the study were explained to the medical students in advance. The researchers also stated that no sensitive information would be passed on, ensuring the anonymity of participants. According to national legislation and the institutional requirements, no confirmation documents or agreements were required to prove the consent of the participants. Participants' behaviors in filling out the questionnaire represented that they agreed to participate in the study. All the questionnaires were filled out with participants' consent in the end. After excluding invalid data, 807 valid questionnaires were collected (339 males, 42.00%; 468 females, 58.00%).

### Measurements

#### Demographic information

The first part of the questionnaire collected demographic information about participants including gender, grade (1st year, 2nd year, 3rd year, 4th year and above), major (clinical medicine or other), as well as basic information about online learning during the COVID-19 pandemic, such as the number of online courses and daily online learning duration.

The second part of the questionnaire, consisting of four 6-point Likert scales (rating from “5” strongly agree to “1” strongly disagree), measured the mental status of medical students in online learning during the 2021 fall semester, including stress, resilience, and academic burnout, as well as social support.

#### Stress scale

The items measuring medical students' stress were selected from the DASS scale developed by Lovibond and Lovibond (56). The reliability and validity of this scale for assessing individuals' depression, anxiety, and stress in Chinese environments has been verified (57), with seven items measuring individual stress. In order to fit the special background of online learning, the items were adjusted, for example, “During the process of online learning, I found it hard to wind down,” “During the process of online learning, I tended to over-react to situations,” “During the process of online learning, I felt that I was using a lot of nervous energy,” and so on. A higher score indicates a greater severity of stress.

#### Social support scale

To measure medical students' perceived social support during the COVID-19 pandemic, the Multidimensional Scale of Perceived Social Support (MSPSS) designed by Gregory et al. (58) was applied. The MSPSS, adjusted for the context of online learning, comprises 12 items, for example, “My family really tries to help me in online learning, such as providing me with online learning equipment, etc.” The higher the total score, the higher the degree of social support that is received by medical students in online learning during the COVID-19 pandemic.

#### Resilience scale

The scale measuring medical students' resilience in online learning during the COVID-19 pandemic was adapted from the 10-Item Connor–Davidson Resilience Scale (CD-RISC) (59). Similarly, all the items were adjusted to fit the context of online learning, for example, “During the process of online learning, coping with stress can strengthen me,” and “During the process of online learning, I am able to adapt to change.” A higher score on this scale indicates a higher level of resilience facing online learning during the COVID-19 pandemic.

#### Academic burnout scale

Medical students' academic burnout was measured using the Maslach Burnout Inventory–Student Survey (MBI-SS) validated by Schaufeli et al. (26). The scale, consisting of 15 items, has been shown to be useful for measuring burnout in groups of students who are exhausted by learning demands. The revised questions are more in line with the characteristics of online learning, for example, “I feel used up at the end of a day of online learning,” and “After online learning, I have become less enthusiastic about my studies.” A higher score indicates a greater degree of academic burnout.

### Item analysis

Item analysis was used to eliminate inappropriate questions from the questionnaire to modify the hypothesized model of this study. At the beginning, there were totally 44 items in the original questionnaire, which included seven items for the Stress Scale, 12 for the Social Support Scale, 10 for the Resilience Scale, and 15 for the Academic Burnout Scale. Firstly, the items with factor loadings below 0.4 were deleted. (60). After this process, four items in the Social Support Scale, one in the Resilience Scale, and five in the Academic Burnout Scale, the factor loadings of which were below 0.4, were deleted. Secondly, first-order confirmatory factor analysis (CFA) was applied to remove inappropriate items from each structure based on residuals until the residuals reached the ideal threshold (61). After conducting CFA (as shown in Table 1), two items in the Stress Scale, four in the Social Support Scale, two in the Resilience Scale, and five in the Academic Burnout Scale were deleted. Finally, 21 items in total remained for further analysis, including five for the Stress scale, four for the Social Support scale, seven for the Resilience scale, and five for the Academic Burnout scale.

**Table 1:** Results of CFA.

**Fit indices**	***x*** ^2^ **/*df***	**GFI**	**AGFI**	**NFI**	**CFI**	**RMSEA**
Result	2.816	0.945	0.930	0.964	0.976	0.047
Threshold	<3.0	>0.9	>0.9	>0.9	>0.9	<0.05

### Reliability and validity analysis

The results of reliability and validity analysis are shown in Table 2. Internal consistency reliability (Cronbach's alpha) exceeded 0.8 for all items, and composite reliability (CR) values for all items ranged from 0.855 to 0.944, indicating good reliability of this study's constructs. (61).

**Table 2:** Results of reliability and validity analysis.

**Variables**	**FL**	**CR**	**AVE**	**Cronbach's** α
Social support	0.573~0.877	0.8548	0.6014	0.842
Stress	0.705~0.925	0.9217	0.7037	0.920
Resilience	0.709~0.888	0.9437	0.7064	0.943
Academic burnout	0.783~0.924	0.9368	0.7485	0.935

The convergent validity and discriminant validity were tested to verify the validity of all the items. As shown in Table 3, the values of both factor loading and average variance extracted (AVE) were higher than 0.5, indicating the acceptable convergent validity of all items. Meanwhile, all the items' square root of AVE were higher than the Pearson correlation values in the off-diagonal constructs. Thus, the discriminant validity of the constructs was suitable.

**Table 3:** Result of discriminant validity analysis.

**Constructs**	**Social support**	**Stress**	**Resilience**	**Academic burnout**
Social support	0.776			
Stress	−0.256	0.839		
Resilience	0.571	−0.417	0.840	
Academic burnout	−0.306	0.738	−0.483	0.865

### Data analysis

Statistical analysis was conducted using SPSS 25.0, including descriptive statistical analysis of demographics and categorical variables, difference analyses of academic burnout in the different groups, and correlation analysis between variables. According to prior studies (1, 62), the results of normality verification do not need to be reported. Then, academic burnout (the dependent variable) was analyzed by hierarchical regression analysis. Demographic characteristics, stress, social support, and resilience were put into the regression equation in steps as follows. Step 1: Demographic characteristics including gender, grade, major, online learning time, number of online courses; Step 2: stress; Step 3: social support; and Step 4: resilience. Finally, structural equation modeling (SEM) analysis conducted using the Amos 26.0 software tested whether resilience mediated the relationship between stress-social support and academic burnout of medical students in online learning during the COVID-19 pandemic. Stress and social support were modeled as independent variables, resilience as a mediating variable, and academic burnout as a dependent variable.

## Results

### Descriptive and difference analysis

Descriptive statistics of demographic characteristics and academic burnout difference in categorical variables are shown in Table 4. Of the 807 medical undergraduates in total, 58% (N = 468) were female and 42% (N = 339) were male. The distribution of grades was even, with 193 (23.9%) in their first year of study, 205 (25.4%) in the second year; 170 (21.1%) in the third year; while 239 (29.6%) were in their fourth year and above. Most of the medical students were from clinical medicine (N = 599, 74.22%). About half of the medical students (N = 432, 53.5%) spent 3~4 h on online learning per day during COVID-19, while 195 (24.2%) spent 1~2 h and 180 (22.3%) spent over 6 h. About half of the medical students (N = 391, 48.4%) took 5~8 online courses, while 312 (38.7%) took 1~4 online courses and 104 (12.9%) took more than 8 online courses. Fourth year students experienced greater severity of academic burnout in online learning than first year students who had just entered college (*p* < 0.01).

**Table 4:** Descriptive analysis and differences in academic burnout among medical students (*N* = 807).

**Variables**	**N**	**%**	**Academic burnout**		
			**Mean**	**SD**	**F**
Gender					5.739
Male	339	42	2.71	0.89	
Female	468	58	2.64	0.76	
Grade					4.208^**^
Freshman	193	23.9	2.53	0.84	
Sophomore	205	25.4	2.62	0.81	
Junior	170	21.1	2.74	0.82	
Senior and above	239	29.6	2.79	0.79	
Major					5.71
Clinical medicine	599	74.22	2.66	0.84	
Others	208	25.78	2.7	0.75	
Online learning time (h)					0.759
1~3	195	24.2	2.65	0.76	
3~6	432	53.5	2.71	0.79	
>6	180	22.3	2.62	0.94	
Numbers of online courses					2.395
1~4	312	38.7	2.6	0.78	
5~8	391	48.4	2.74	0.79	
>8	104	12.9	2.66	1.03	

** Significant at the 0.01 level.

### Correlative analysis

The results of means, standard deviations of social support, stress, resilience, and academic burnout as well as the correlative analysis are demonstrated in Table 5. Both social support and resilience were significantly and negatively correlated with medical students' academic burnout in online learning (*p* < 0.01), while stress was significantly and positively correlated with academic burnout (*p* < 0.01) among medical students in online learning.

**Table 5:** Result of the correlative analysis.

	**Mean**	**SD**	**1**	**2**	**3**	**4**
1. Social support	3.65	0.69	1			
2. Stress	2.67	0.78	−0.256^**^	1		
3. Resilience	3.55	0.65	0.571^**^	−0.417^**^	1	
4. Academic burnout	2.67	0.82	−0.306^**^	0.738^**^	−0.483^**^	1

** Significant at the 0.01 level.

### Hierarchical linear regression analysis

Academic burnout was regarded as the dependent variable, and demographics including gender, grade, major, online learning time, and number of online courses were treated as the control variable. Stress and social support as independent variables were introduced into the hierarchical linear regression analysis.

#### Stress, resilience, and academic burnout

Stress and resilience were successively introduced into the regression model. As shown in Table 6, stress was a significant predictor of academic burnout, accounting for 54.9% of variation. When resilience was added to the model, the standardized regression coefficient (β) between stress and academic burnout decreased from 0.733 to 0.646. This result indicated that resilience might play a partial mediating role in the relationship between stress and academic burnout among medical students in online learning during the COVID-19 pandemic.

**Table 6:** Hierarchical linear regression analysis (stress as independent).

**Block**		**Academic burnout**		
		**Model 1**	**Model 2**	**Model 3**
1	Demographics			
	Gender	−0.052	−0.011	−0.018
	Grade	0.132^**^	0.063^**^	0.038
	Major	−0.018	−0.012	−0.033
	Online learning time	−0.055	−0.023	0.006
	Number of online courses	0.038	−0.002	−0.001
2	Stress		0.732^**^	0.646^**^
33	resilience			−0.213^**^
R^2^	0.020	0.549	0.585
Δ*R^2^*	0.014	0.546	0.581

** Significant at the 0.01 level.

#### Social support, resilience and academic burnout

Social support was also regarded as an independent variable to carry out the hierarchical linear regression analysis. The results are shown in Table 7. After controlling for gender, grade, major, online learning time, and number of online courses, social support predicted academic burnout in online learning among medical students with an explanatory variance of 9.8%. When resilience was introduced into the model, it made a new contribution and increased the explanatory variation of academic burnout by 14%. However, the standardized regression coefficient of stress to depression decreased from 0.294 to 0.039 (*p* > 0.05), which suggested that resilience may completely mediate the impact of social support on academic burnout.

**Table 7:** Hierarchical linear regression analysis (social support as independent).

**Block**		**Academic burnout**		
		**Model 1**	**Model 2**	**Model 3**
1	Demographics			
	Gender	−0.052	−0.039	−0.054
	Grade	0.132^**^	0.101^**^	0.056
	Major	−0.018	−0.034	−0.062
	Online learning time	−0.055	−0.027	0.020
	Number of online courses	0.038	0.030	0.029
2	Social support		−0.294^**^	−0.039
33	resilience			−0.464^**^
R^2^		0.020	0.105	0.245
Δ*R*^2^		0.014	0.098	0.238

** Significant at the 0.01 level.

### Structural equation modeling of the mediating role of resilience

**Figure 2:**
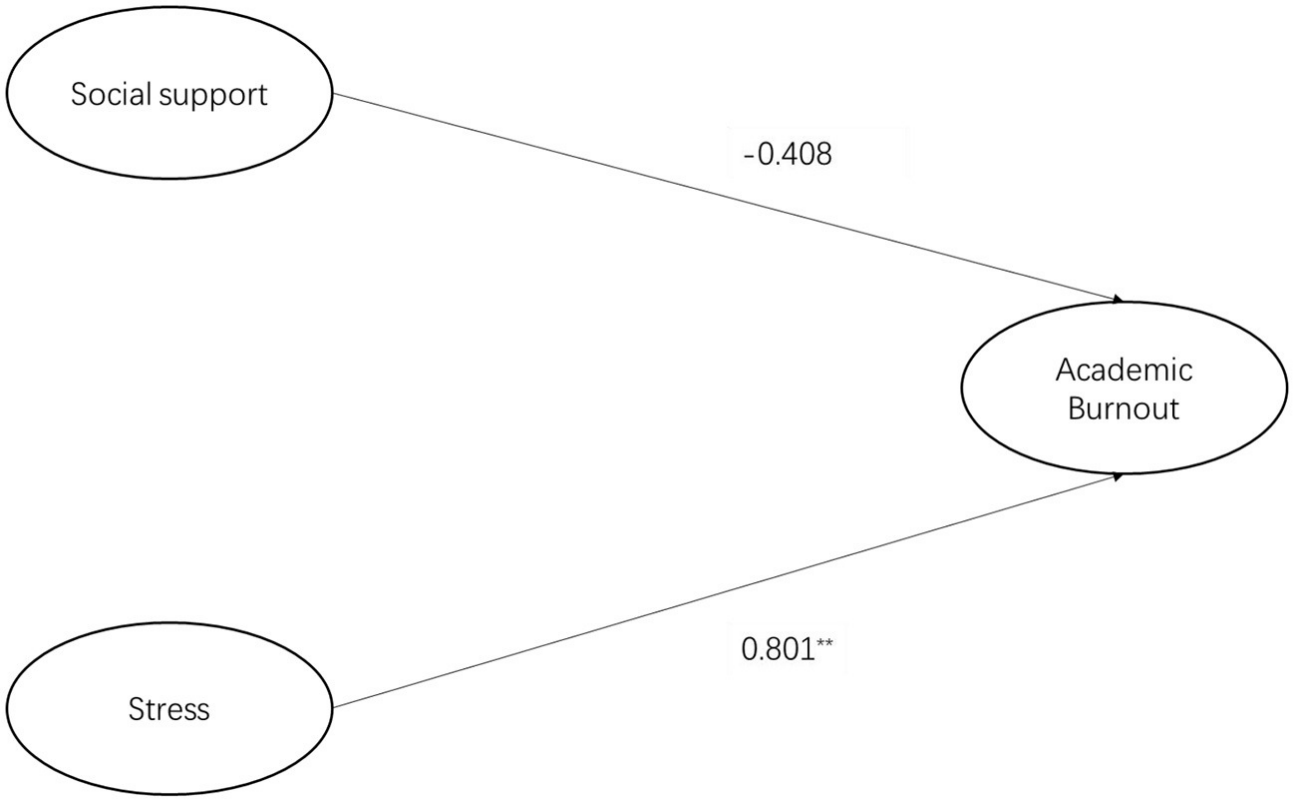
SEM of stress, social support, and academic burnout.

**Figure 3:**
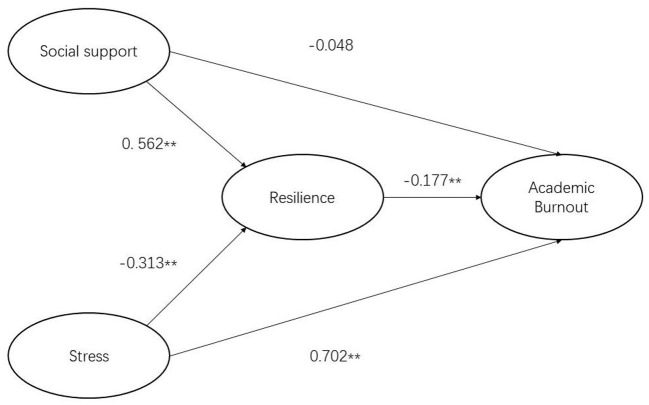
SEM of the mediating role of resilience between stress, social support, and academic burnout.

The results of structural equation modeling analysis conducted using Amos 26.0 are presented in Table 8. The observed data fit well with the proposed structural model, which indicated that resilience not only had a direct influence on academic burnout, but also significantly and indirectly affected medical students' academic burnout in online learning *via* stress and social support. Firstly, the direct pathways from stress and social support to academic burnout are illustrated in Figure 2. Stress had a significant and positive influence on academic burnout (β = 0.801, *p* < 0.01; H1a supported), while social support had no effect on academic burnout (β = −0.408, *p* > 0.05; H1b rejected). Figure 3 illustrates the indirect pathways from stress and social support to academic burnout *via* resilience. As is shown, stress was negatively associated with resilience (β = −0.313, *p* < 0.01; H2a supported), social support was positively associated with resilience (β = 0.562, *p* < 0.01; H2b supported), and resilience had a negative association with academic burnout (β = −0.177, *p* < 0.01; H3 supported). The bootstrap and bias-corrected method was employed to test the indirect effect of resilience. As shown in Table 9, resilience played a mediating role between stress and academic burnout (*p* < 0.01, a ^*^ b = 0.056, H4a accepted), as well as social support and academic burnout (*p* < 0.01, a ^*^ b = −0.100, H4b accepted). In combination with the indirect effect and the direct effect, it can be concluded that resilience partially mediated the relation between stress and academic burnout, while resilience completely mediated the relation between social support and academic burnout.

**Table 8:** Result of SEM analysis.

**Fit indices**	*x* ^2^ **/*df***	**GFI**	**AGFI**	**NFI**	**CFI**	**RMSEA**
Result	2.816	0.945	0.930	0.964	0.976	0.047
Threshold	<3.0	>0.9	>0.9	>0.9	>0.9	<0.05

**Table 9:** Indirect effect of the model.

**Pathway**	β	**se**	**Bias-corrected 95% CI**	
			**LL**	**UL**
Stress → Resilience → AB	0.056^**^	0.018	0.026	0.096
Social support → Resilience → AB	−0.100^**^	0.026	−0.156	−0.054

** Significant at the 0.01 level.

## Discussion

The purpose of this study was to explore the relationship between stress, social support, resilience, and academic burnout among medical students in online learning during the COVID-19 pandemic, and to examine the mediating effect of resilience on stress-social support and academic burnout. To achieve this objective, this study provided empirical evidence in the relationship between stress, social support, resilience, and academic burnout based on relative theoretical concepts and conclusions from prior research on medical students. This study fills a gap in the existing literature.

Firstly, the results of this study indicate that stress and resilience positively predicted academic burnout, and social support insignificantly predicted academic burnout. That is, stress might be a risk predictor of academic burnout, while resilience might be a protective predictor of academic burnout. Secondly, as expected, the relationship between stress and academic burnout was partially mediated by resilience among medical students in online learning, while resilience completely mediated the relation between social support and academic burnout. The findings are described and explained in more detail below.

### The correlation between stress, social support, resilience and academic burnout

#### Stress: Positively related to academic burnout but negatively related to resilience

Compared with students from other majors, the problem of stress is more prominent among medical students (63, 64). During the COVID-19 pandemic, medical students have had to acquire knowledge through online courses, which has put enormous stress on them in terms of both their academic study and opportunities of employment (65). Results of this study indicated that stress has a positive effect on academic burnout in the online learning process of medical students, indicating that it is one of the important risk factors of academic burnout, and the occurrence of academic burnout should be avoided by regulating students' stress in online learning; thus, H1a was supported. This finding was in line with prior studies which confirmed that stress was interlinked with academic burnout among medical students [e.g., (66–68)]. Guruprakash et al. (66) pointed out that after experiencing great stress, medical students would show painful emotions and have relatively higher scores for burnout. Specifically, the Job Demands and Resources (JD-R) model proposed that the increase in job demands (stress) can lead to job burnout (53). He et al. (69) also confirmed that stress as one of the job demands was positively related to clients' burnout on the basis of the central postulates of the Job Demands and Resources (JD-R) model. Due to the sudden changes in online learning during the COVID-19 pandemic, medical students have reported higher levels of confusion and stress than before (70, 71). During the period of the COVID-19 pandemic, the level of medical students' stress was inevitably increased as they had to study online at home, which would accordingly result in negative effects on their study (72); academic burnout should be one of the adverse reactions.

In addition to having a negative impact on medical students' online learning, especially aggravating their academic burnout, stress will also cause mental health problems. This study found that there was a negative correlation between stress and resilience of medical students in online learning during the COVID-19 pandemic; thus, H2a was supported. This finding is consistent with the research of Yu et al. (73) which indicated that lower stress during COVID-19 was significantly associated with higher resilience at the time of admission.

Therefore, it is necessary to provide students with effective strategies and coping methods to manage and reduce stress in the post-COVID-19 pandemic era. Studies have shown that self-care activities such as exercise, healthy diet, and building good interaction with others can buffer stress (74), while qualities such as self-efficacy, happiness, and optimism can alleviate stress (75).

#### Social support: Unrelated to academic burnout but positively related to resilience

Specifically, Santen et al. (27) confirmed that the low level of support and high stress resulted in a high degree of burnout which gradually developed in the course of medical education. Social support is important for helping medical students overcome academic burnout in most situations, as has been confirmed in most literature. However, these views deviate from another finding of this study, that is, social support, differing from stress, was not a significant predictor of medical students' academic burnout in online learning, and thus H1b was rejected. Social support can effectively reduce the risk of academic burnout based on the precondition of subjective support and learners' use of support (76). Fontana et al. (77) also implied that social support seeking behavior did not effectively reduce the prevalence of burnout among medical interns. Through a systematic literature review, Chunming et al. (78) concluded that social factors such as the degree of social support, or the environmental factors around students jointly affect the burnout of medical students. Furthermore, García-Sierra et al. (54) found that differing from job demands which were significant predictors of nurses' burnout, social support significantly predicted nurses' engagement according to the Job Demands and Resources (JD-R) model. Therefore, social support might not independently alleviate the symptoms of academic burnout in medical students in the context of online learning.

However, the positive relationship between social support and medical students' resilience in online learning was found in this study, and thus H2b was supported. This finding is in accordance with previous literature on the correlation between social support and resilience. Casapulla et al.'s (79) research indicated that social support perceived or experienced by medical students is an important factor in their progress toward resilience. Bore et al. (80) also provided evidence that social support positively correlated with medical students' social support. When dealing with a crisis such as the COVID-19 pandemic, effective social support can alleviate negative emotions, improve self-efficacy, and build up confidence and courage to cope with the crisis (81).

Therefore, providing the necessary and proper social support services for medical students who experience high levels of burnout and low levels of resilience (82) during the online learning of the COVID-19 pandemic is crucial, as it might maximize the resilience level of medical students to some extent. For example, schools can offer online psychological counseling services or online courses on mental preparation for public health events for medical students (11).

#### Resilience: Negatively related to academic burnout

In addition to finding that increased social support and decreased stress can prevent the symptom of burnout, resilience is also an important strategy to diminish burnout in this study; thus, H3 was supported. Previous studies on the correlation between resilience and academic burnout among medical students indicated similar results as this study. Pharasi and Patra (83) concluded that resilience is a protective mechanism against burnout. Through the investigation of medical students, Forycka et al. (82) reported that students with a higher level of resilience presented a better attitude toward online courses and showed lower levels of academic burnout.

Zuniga et al. (84) noted that teaching self-awareness, formal educational interventions, and self-regulation skills can help improve resilience and promote wellbeing, even during a pandemic. Dunn et al. (85) developed a special model called the “Coping Reservoir” to promote resilience in medical students to combat burnout symptoms. Through a survey of medical students who had completed long-term resilience skills training, Mugford et al. (86) concluded that planned rest time, establishment of a support system, and mindfulness skills were all effective measures to train resilience.

### The partial mediating role of resilience in stress and academic burnout

As expected, the relationship between stress and academic burnout was partially mediated by resilience among medical students in online learning; thus, H4a was supported. This finding is in concordance with Farquhar et al. (87), who pointed out that resilience has a preventive effect on burnout because it can reduce medical students' perception of stress. In the literature on the stress of medical students, resilience training is also regarded as an effective factor to relieve the impact of stress (88). Duarte et al. (89) concluded that resilience played a mediating role in perceived stress and burnout (exhaustion) among medical students during the COVID-19 lockdown. Resilience is considered necessary for medical students to overcome the stress from academic and future employment and to achieve academic success (90). Through an empirical investigation of a large sample of medical students, Peng et al. (91) concluded that resilience can greatly mitigate the impact of mental health problems on medical students and help students adapt to negative life events. The COVID-19 pandemic, which has forced students to study online at home and has disconnected them from school and practice sites, is a negative life event for medical students without a doubt. Intermittent online learning during the COVID-19 pandemic period has aggravated the stress of medical students, and academic burnout is one of the chain reactions of stress. Resilience, as a mediator of medical students' stressors, has positive and far-reaching significance for the development of medical students (92). Therefore, resilience strategies should be proposed to mediate the negative correlation between stress and burnout among medical students.

Learning coping skills of adapting to stress is an effective measure to strengthen resilience indicators and to reduce the negative effects of stress among medical students (93). Building reliable resilience of students also needs the assistance of teachers. Faculty strategies for decreasing stress and increasing resilience among medical students has been presented in prior studies (87). It is feasible to communicate negative emotions related to stress with students, as well as to share teachers' experiences of stress and resilience through structured activities such as lectures and conferences. In addition, teachers can guide students to maintain positive mental imagery when facing stress.

### The complete mediating role of resilience in social support and academic burnout

Although the direct effect of social support on academic burnout was not found, resilience played a complete mediating role in the relation between social support and academic burnout among medical students in online learning during the COVID-19 pandemic. This finding is consistent with the research of Klinoff et al. (94), which revealed that the significant association between social support and burnout was mediated by resilience. Similarly, Shang and Yang (95) demonstrated that social support received by athletes inhibits or prevents the prevalence of burnout through psychological resilience. The social support caused by the isolation from society does not directly cause academic burnout of medical students in the COVID-19 pandemic, but rather the structure that poor social support leads to low resilience causes the aggravation of academic burnout among medical students in the process of online learning. Therefore, when providing social support policies for medical students, more attention should be paid to enhancing their resilience relying on social support, so as to compensate for the symptoms of academic burnout.

## Conclusions

During the continuing COVID-19 pandemic, online teaching was introduced as an important supplement to regular teaching. Online learning and the COVID-19 lockdown have led to a sharp decrease in social support for medical students and an increase in stress, which requires resilience to mitigate the swelling of academic burnout caused by the sudden decrease in social support and the surge in stress. The conclusions of this study provide some suggestions for stress relief, social support provision, resilience development, and academic burnout mitigation for medical students in future online learning.

The results of this study demonstrated that stress exhibits a direct positive effect on academic burnout and an indirect effect on academic burnout through resilience, whereas social support has no direct effect on academic burnout, but exerts an indirect effect on academic burnout through the mediation path of resilience. Therefore, it is suggested that intervention measures be provided to reduce the academic burnout of medical students in online learning during the COVID-19 pandemic, especially focusing on the stress and resilience of medical students, and providing strategies for enhancing resilience while increasing social support to better reduce academic burnout of medical students.

### Implications

The implications of this research for society or practice are presented below from theoretical and practical perspectives.

The theoretical contributions of this study are as follows. Firstly, this study has enriched the literature on stress, social support, resilience, and academic burnout among medical students. In the past, the research on medical students' mental health or academic performance was mostly confined to the traditional face-to-face learning environment or internship background. There are few studies on the relationship between stress, social support, resilience, and academic burnout in the context of online learning.

Secondly, in this study, stress (the negative predictor) and social support (the positive predictor) were used as independent variables to explore the variation of medical students' academic burnout, and two mediation path models with resilience as the mediator were constructed. Different from previous mediating models that considered only one independent variable, this study better demonstrates the buffering effect of resilience on the impact of positive and negative factors on academic burnout.

Finally, distancing measures taken during the COVID-19 pandemic have imposed unprecedented restrictions on medical students' learning. Medical students' stress, perceived social support, and resilience levels were considered as predictors of academic burnout over the years. Therefore, it is of great significance to explore the prevalence of academic burnout among medical students in online learning for the continuation of medical education.

The theoretical model of this study also has practical contributions which might be used in practice and teaching. The biggest practical contribution of this study is that it identifies the appropriate intervention measures for mental health maintenance and academic burnout offsetting for multiple levels of medical students, and for teachers and education departments. These interventions will help reduce the risk of stress and academic burnout, and enhance the sense of perceiving social support and more stable resilience among medical students.

This research was conducted in the fall semester of 2021, when most Chinese universities had accumulated some experience of online teaching and students had already conducted several rounds of online learning at home. Therefore, compared with the research on online teaching carried out in the early stage of the COVID-19 pandemic, the conclusion of this study may provide more valuable references for other countries to carry out online teaching in the future.

#### Limitations and future study

One limitation of the study is the adoption of a cross-sectional design. Although statistical methods have been used to explore a causal relationship between stress, social support, resilience, and academic burnout, the explanation is not sufficient. Therefore, it is necessary to conduct longitudinal studies on similar cohorts in the future to further explore their internal associations.

Another limitation of this study is the quantitative analysis of the self-assessment tools used in this study to measure the indicators of stress, social support, resilience, and burnout of medical students. In the future, a mix of quantitative and qualitative approaches can be designed to present integrated and individual experiences and ideas in greater detail and to make possible solutions and suggestions more reasonable.

## Data availability statement

The original contributions presented in the study are included in the article/supplementary material, further inquiries can be directed to the corresponding author.

## Ethics statement

The studies involving human participants were reviewed and approved by the Ethics Committee of the Second Affiliated Hospital of Nanjing Medical University. Written informed consent for participation was not required for this study in accordance with the national legislation and the institutional requirements.

## Author contributions

All authors contributed equally to the conception of the idea, implementing and analyzing the experimental results, writing the manuscript, and reading and approving the final version of the manuscript.

## Conflict of interest

The authors declare that the research was conducted in the absence of any commercial or financial relationships that could be construed as a potential conflict of interest.

## Publisher's note

All claims expressed in this article are solely those of the authors and do not necessarily represent those of their affiliated organizations, or those of the publisher, the editors and the reviewers. Any product that may be evaluated in this article, or claim that may be made by its manufacturer, is not guaranteed or endorsed by the publisher.
